# Case Report: Cracking the case: unique presentation of pulmonary sarcomatoid carcinoma with spherical lung metastases

**DOI:** 10.3389/fmed.2025.1567046

**Published:** 2025-04-25

**Authors:** Qiu-Lian Liu, Kexin Cao, Jun-Da Cao, Yu Zhang, Bang-Ming Guo

**Affiliations:** ^1^Jiujiang City Key Laboratory of Cell Therapy, Department of Oncology, The First Hospital of Jiujiang City, Jiujiang, China; ^2^Department of Thoracic Surgery, The First Affiliated Hospital of Xinxiang Medical University, Xinxiang, China; ^3^Jiujiang City Key Laboratory of Cell Therapy, Department of Cardiovascular Medicine, The First Hospital of Jiujiang City, Jiujiang, China; ^4^Department of Pathology, General Hospital of Central Theater Command of the Chinese People’s Liberation Army, Wuhan, China; ^5^Department of Neurosurgery, First Affiliated Hospital of Gannan Medical University, Ganzhou, China

**Keywords:** multiple masses, sarcomatoid carcinoma, metastases, lung cancer, differential diagnosis

## Abstract

**Background:**

Pulmonary sarcomatoid carcinoma (PSC) is rare among lung malignancies, but it has a high degree of malignancy, and the average survival time for clinically diagnosed patients with PSC is less than 1 year. There are few reports of multiple solid spherical metastases in the lungs of patients with PSC.

**Case presentation:**

This case presents an 80-year-old male with a history of Alzheimer’s disease who was hospitalized due to a frequent dry cough. A 16-slice computed tomography examination of the chest revealed multiple solid, egg-sized lesions with smooth borders in both lungs. Because this type of medical imaging data is very rare in clinical practice, the patient’s diagnosis was initially confusing. Given that the patient had no history of fever and no special abnormalities in the results of laboratory tests, the multidisciplinary team (MDT) suggested that lung metastases or lung malignancies with intrapulmonary metastasis should be considered. A magnetic resonance imaging (MRI) examination of the head was performed and revealed brain metastatic lesion. Finally, a puncture biopsy of the lung lesion was performed under ultrasound guidance. The case results confirmed PSC.

**Conclusion:**

This case presents a very rare medical imaging dataset of solid lesions with multiple egg-sized having smooth borders in both lungs. This special medical imaging data highlights the variability of PSC imaging and the complexity of accurate diagnosis, aiming to improve the understanding of PSC.

## Introduction

Multiple solid lesions in both lungs can present a diagnostic challenge due to their rarity and the broad differential diagnoses they encompass (1–3). These lesions may represent a variety of pathologies, including metastatic disease, multiple primary lung cancers, infectious processes, or benign conditions. For example, tuberculosis and non-tuberculous infectious lesions can appear as solid lung nodules or masses. Understanding the underlying cause is crucial for determining the appropriate management and prognosis. Currently, the presence of multiple solid lesions in both lungs necessitate a thorough diagnostic workup, including imaging studies and possibly histopathological examination, to identify the underlying cause and guide appropriate treatment strategies. Previous reports have shown multiple metastases in the lungs of patients with pulmonary sarcomatoid carcinoma (PSC) (4). However, there are no published reports on PSC that include a medical imaging dataset of solid lesions with multiple egg-sized nodules having smooth borders in both lungs. This case presents an 80-year-old male with this very rare medical imaging dataset.

## Case report

An 80-year-old male was hospitalized with an irritating dry cough that had persisted for 2 weeks. This cough was unaffected by temperature and pungent odors. The patient was generally in good health except for Alzheimer’s disease. His Alzheimer’s disease mainly manifested as forgetfulness, dementia, and lethargy, but he believed that he could take care of himself. Consequently, he refused medication for Alzheimer’s. The patient and his companion denied that he had asthma, chronic obstructive pulmonary disease, or gastroesophageal reflux disease. He did not exhibit any signs of distress or chest discomfort and was able to climb four flights of stairs without requiring rest. He recently denied having a fever, obvious night sweats, low-grade fever, skin-related abnormalities, diarrhea, or edema in his lower extremities. Meanwhile, he also stated that he had no sputum production, chest tightness, dyspnea, night sweats, cyanosis, or clubbing of the fingers. The patient is currently eating normally, and his food intake has not changed significantly in the past 10 years. However, he has lost about 3 kg in the last 3 months. The patient has been smoking for more than 60 years and has not quit, with a daily average of 20 cigarettes. He has 5 years of prior work experience with asbestos exposure. According to the patient’s companion, the chest X-ray examination conducted after the end of that work did not show significant abnormalities. The patient has no history of pet ownership or unclean sexual contact. His professional occupation was not related to any special exposure or radiation exposure, and he reported no alcohol consumption, illicit drug use, or recent travel.

**Figure 1:**
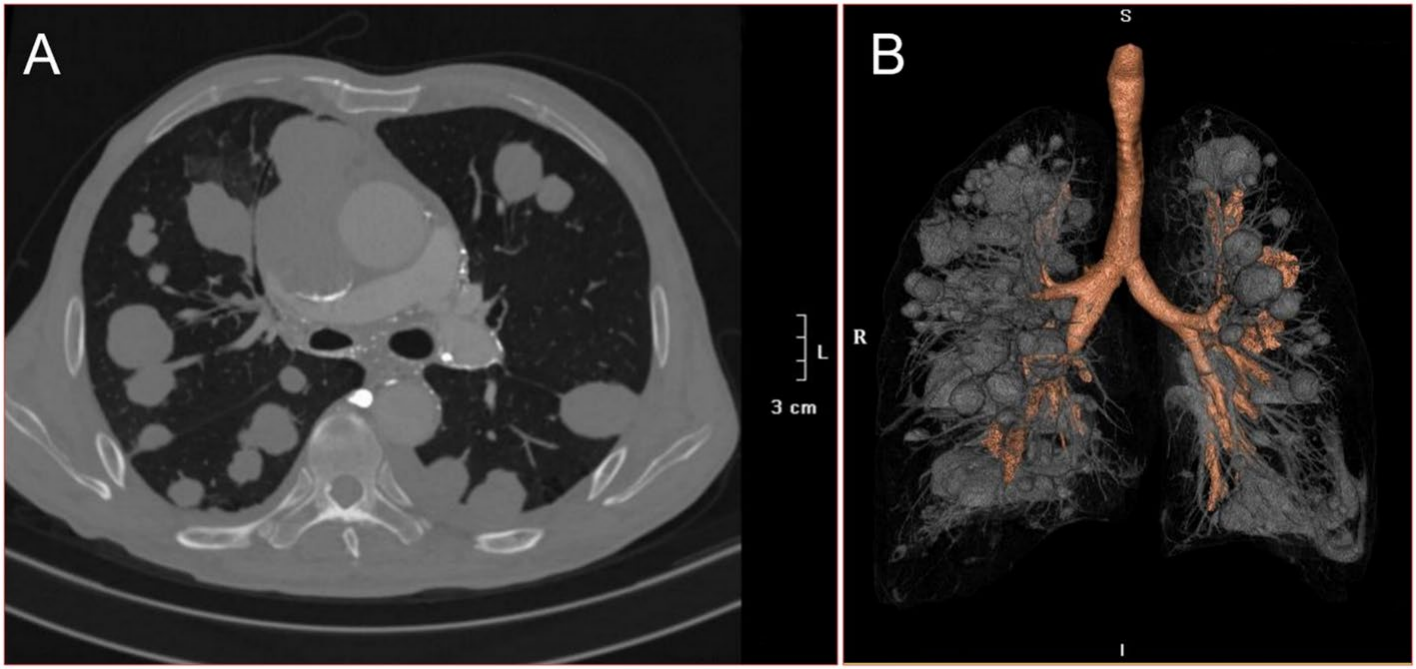
16-slice computerized tomography images of the chest with the patient at admission. **(A)** Horizontal planes. **(B)** Three dimensional reconstruction.

His physical examination found no obvious bilateral swelling of the supraclavicular lymph nodes, slightly weak bilateral lung breath sounds, scattered wet rales, and pleural friction rubs. He underwent a chest and abdomen computed tomography examination, which revealed multiple solid, egg-sized lesions with smooth borders in both lungs (Figures 1A,B). His laboratory results (complete blood cell count, liver panel, kidney panel, tumor marker) were within normal limits, and his lung function tests were also within normal limits.

Because this type of medical imaging data is very rare in clinical practice, the patient’s diagnosis was initially confusing. One potential cause of multiple solid lesions in the lungs is synchronous multiple primary non-small cell lung cancer (SMPNSCLC). Another possible cause of multiple solid lesions in the lungs is intrapulmonary metastases, such as those from osteosarcoma. In addition to metastatic and primary lung cancers, infectious diseases can also present as multiple solid lesions in the lungs. For example, tuberculosis and invasive candidiasis can manifest as multiple pulmonary lesions.

**Figure 2:**
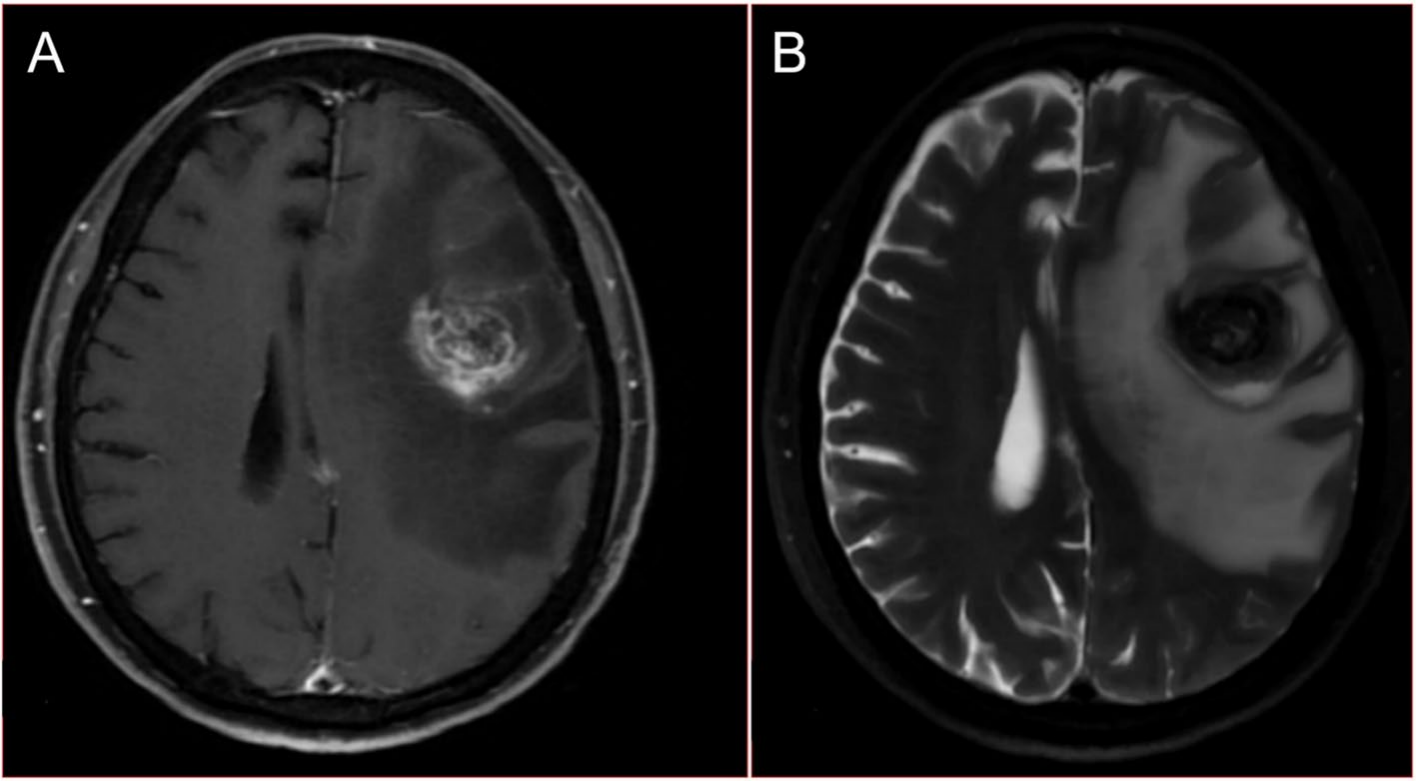
Magnetic resonance images of the head with the patient at admission. **(A)** Contrast enhanced T1WI. **(B)** T2WI.

**Figure 3:**
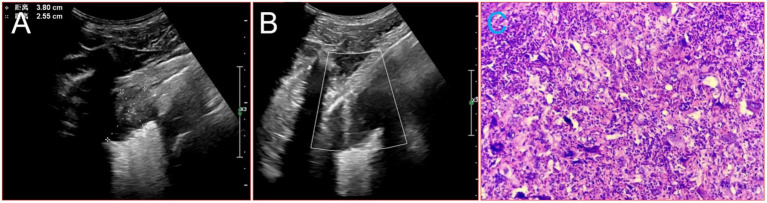
Image about puncture biopsy of the lung lesion under ultrasound guidance, and the histologic results. **(A)** Ultrasound images of the lesion in the patient’s lower lobe of the left lung. **(B)** The process of puncture biopsy of the lung lesion under ultrasound guidance. **(C)** HE × 20.

Given that the patient had no history of fever and no special abnormalities in the results of laboratory tests, the multidisciplinary team (MDT) suggested that lung metastases or lung malignancies with intrapulmonary metastasis should be considered. A magnetic resonance imaging (MRI) examination of the head was performed and revealed brain metastatic lesion (Figures 2A,B). Unfortunately, due to financial constraints, the patient did not undergo the PET-CT examination. At the same time, he underwent gastroscopy and colonoscopy because of concerns about weight loss, but no tumor was found. Finally, a puncture biopsy of the lung lesion was performed under ultrasound guidance, and the histologic results confirmed PSC with giant-cell carcinoma (Figures 3A–C), PD-L1 (=1%, Clone: 22C3, Dako, Agilent Technologies, Inc.).

The actionability of genomic alterations and the level of evidence were determined based on the OncoKB dataset. Drug approval status in mainland China revealed no mutated genes such as EGFR (Exons 18/19/20/21/T790), FGFR2, FGFR3, ROS1, PIK3CA (Exons 10/12, coding exons 9/20), KRAS (Codons 12/13/61/146), TP53, RET, ALK, NRAS (Codons 12/13/61), and KIT (Exons 9/11). Genetic testing was conducted in a CLIA-certified lab using hybridization capture-based next-generation sequencing panels. Gene targets included those from MyGene, BGI-Shenzhen (Headquarters: Shenzhen, 518083, China). The genomic alterations assessed included single nucleotide variations, insertions and deletions, copy number variations, and gene rearrangements in selected genes. The MyGene panels covered common lung cancer-related genes, with a minimum coverage across samples of ≥1,000×.

**Figure 4:**
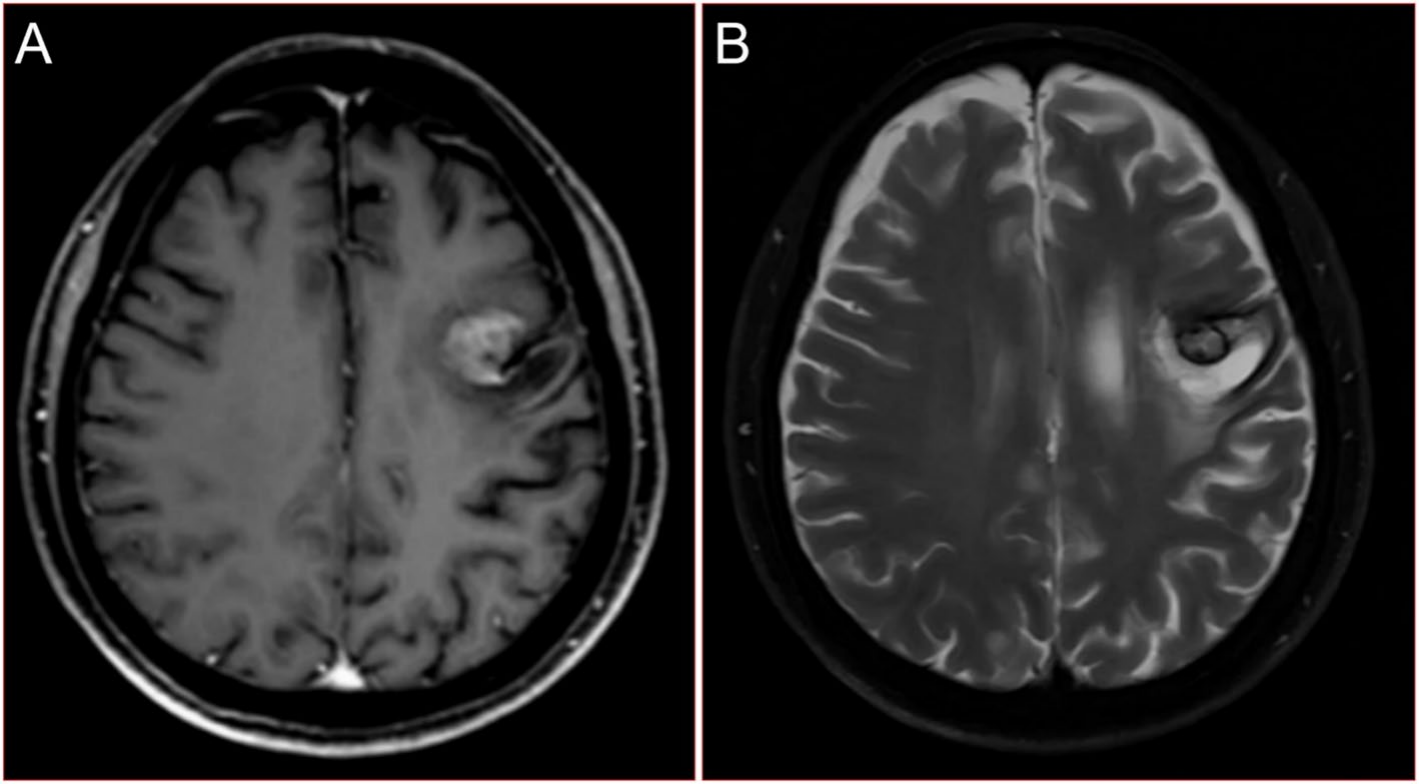
Reexamination magnetic resonance images of the head after chemotherapy of reexamination of the patient. **(A)** Contrast enhanced T1WI. **(B)** T2WI.

He received a diagnosis of stage IVb PSC, cT4N3M1, with a negative EGFR status (as per the ninth edition TNM classification, the presence of numerous lesions in the same lung lobe is classified as T4, while the presence of nodules in the opposite lung lobe and multiple metastases in distant organs is categorized as M1. Additionally, the presence of bilateral hilar and mediastinal lymphadenopathy is classified as N3). He received recombinant human endostatin (Endostar, days 1–14: 7.5 mg/m^2^ every day) combined with pemetrexed (250 mg/m^2^ on day 1) and cisplatin (15 mg/m^2^, daily from days 1 to 3). During this treatment, the patient experienced some common side effects, such as nausea, fatigue, infections, vomiting, decreased appetite, decreased red blood cell count, decreased platelet count, and decreases in the components of white blood cells, which were successfully alleviated with conventional methods. After two cycles of chemotherapy, there was no significant improvement in his symptoms. Reexamination of the head MRI showed that the metastatic tumor had become smaller (Figures 4A,B), but the chest X-ray showed no significant improvement in the lung lesions. Then he encountered a new wave of the COVID-19 pandemic. When he was taken to the hospital after being infected, his face was blue, and he had severe breathing difficulties. A blood oxygen saturation test showed that his peripheral blood oxygen saturation was only 72%. The doctor recommended ventilator treatment with endotracheal intubation, but the patient’s family refused to allow it, citing the patient’s prior wishes. Unfortunately, he died that day. His family refused further autopsy.

## Discussion

PSC is indeed a rare and aggressive subtype of non-small cell lung cancer (NSCLC) that poses significant challenges in imaging diagnosis due to its rarity and the overlap of its morphological features (5, 6). Imaging diagnosis of lung lesion(s) diseases, including artificial intelligence assistance, is difficult to obtain satisfactory accuracy (7). Multiple lesions in lung includes lumpy lesions, nodular lesions and miliary lesions.

Lumpy lesions characterized by several or uncountable discrete pulmonary mass lesions, most were solid lesions, usually larger than 3 cm in diameter, and with or without cavity formation. It can often be associated with metastatic diseases, such as gastrointestinal stromal tumors (GISTs), which are known to metastasize to the lungs, albeit infrequently (8). Other include sarcomatous lung metastasis and malignant melanoma lung metastasis (9, 10), etc. In a few cases, synchronous multiple primary lung cancers, infectious or inflammatory processes can also exhibit this form (11–13).

Nodular lesions are another common form of pulmonary abnormality characterized by uncountable discrete pulmonary nodules lesions usually, most were ground-glass opacity lesions, the diameter varies from 0.5 cm to 3 cm, and accompanied or accompanied by a burr sign. It can be indicative of a range of conditions, from benign to malignant. In this context, two particular diseases deserve attention, primary pulmonary lymphoma and pulmonary cryptococcosis (14, 15). Primary pulmonary lymphoma, which can coexist with other benign lesions such as hamartomas (14). This rare occurrence can mimic invasive pulmonary mycosis, making accurate diagnosis challenging (15). Additionally, benign metastasizing leiomyoma (BML) and pulmonary metastasis can present as multiple nodules in the lungs also (16, 17).

Miliary lesions, characterized by uncountable discrete pulmonary micronodules, generally tiny and uniform in size (≤5 mm) and diffusely distributed throughout the lungs, can represent a wide variety of pathologies, including but not limited to tuberculosis (TB), sarcoidosis, histoplasmosis, coccidioidomycosis, silicosis, malignancies (thyroid, renal, prostate, and breast cancer), etc. In endemic areas, TB is a major cause of miliary nodules (18–21). As the presence of multiple lesions in lung patterns on radiological images is not specific, the final diagnosis cannot be established without a tissue biopsy.

This type of carcinoma is characterized by its poor prognosis and resistance to conventional treatments, making it a subject of ongoing research and clinical interest (22–25). Due to its rarity and aggressive nature, the treatment options for PSC are limited and often result in suboptimal outcomes. However, recent advancements in targeted therapies and immunotherapies have shown promise in improving the prognosis for patients with PSC. Studies have shown that PSC often expresses high levels of PD-L1, making it a potential candidate for treatment with PD-1/PD-L1 inhibitors (26–28). In addition to immunotherapy, targeted therapies have also been explored for PSC. The presence of MET exon 14 skipping mutations in some PSC patients provides an opportunity for targeted treatment (28). Furthermore, the identification of novel genetic alterations, such as the PHF20-NTRK1 fusion, in PSC patients opens up possibilities for targeted treatment with NTRK1 inhibitors, which could potentially improve outcomes for patients with these specific genetic profiles (29). Despite these advancements, the overall prognosis for PSC remains poor, and there is a need for further research to develop more effective treatment strategies (30). The combination of immunotherapy with other treatment modalities, such as radiotherapy, has shown potential in enhancing treatment efficacy and warrants further investigation (31).

In conclusion, there are no published reports on PSC with multiple synchronous lung and brain metastases, this case presents a very rare medical imaging dataset of solid lesions with multiple egg-sized having smooth borders in both lungs. This special medical imaging data highlights the variability of PSC imaging and the complexity of accurate diagnosis, aiming to stimulate discussion of the disease, and to gain more attention for targeted diagnosis and treatment.

## Data Availability

The original contributions presented in the study are included in the article/supplementary material, further inquiries can be directed to the corresponding authors.
